# Tobacco use in Lao People’s Democratic Republic: Results from the 2015 National Adult Tobacco Survey

**DOI:** 10.18332/tpc/112248

**Published:** 2019-09-30

**Authors:** Phonepadith Xangsayarath, Daovieng Douangvichith, Latsamy Siengsounthone, Khatthanaphone Phandouangsy, Ly Thi-Hai Tran, Thanh Cong Bui

**Affiliations:** 1 National Center for Laboratory and Epidemiology, Ministry of Health, Vientiane, Lao PDR; 2 Ministry of Health, Vientiane, Lao PDR; 3 Lao Tropical and Public Health Institute, Ministry of Health, Vientiane, Lao PDR; 4 Department of Hygiene and Health Promotion, Ministry of Health, Vientiane, Lao PDR; 5 Oklahoma Medical Research Foundation, Oklahoma, United States; 6 Department of Family and Preventive Medicine, Oklahoma Tobacco Research Center, Stephenson Cancer Center, University of Oklahoma Health Sciences Center, Oklahoma, United States

**Keywords:** cigarette smoking, tobacco use, Lao People’s Democratic Republic

## Abstract

**INTRODUCTION:**

Tobacco use is a burden for Lao People’s Democratic Republic (Lao PDR). No published report has examined determinants of various tobacco uses to inform appropriate policies and prevention strategies. This paper reports tobacco uses by sociodemographic characteristics using data from the most recent Lao National Adult Tobacco Survey (NATS) in 2015.

**METHODS:**

The NATS included a nationally representative sample of 7562 people aged ≥15 years, recruited through a stratified 2-stage cluster sampling approach in 18 provinces. All analyses were weighted. Multivariable logistic regression models were used to evaluate unadjusted and adjusted associations between variables of interest.

**RESULTS:**

The NATS results showed that 32.4% of Lao people aged ≥15 years were current tobacco users (men: 51.2%, women: 15.4%). Cigarette smoking accounted for approximately 95% of all tobacco use in men, while tobacco chewing accounted for 60% of tobacco use in women. Current tobacco use was strongly associated with older ages and lower education levels (p<0.001). There were interactions between sex, education level, and income associated with tobacco use; specifically, women were more likely to have a lower education level and lower income than men, and these women were more likely to use tobacco.

**CONCLUSIONS:**

Tobacco use prevalence in Lao PDR was among the highest in the region. There were variations in types and prevalence of tobacco use across sociodemographic subpopulations. The Lao government should continue current national tobacco control efforts and implement additional proven strategies to reduce tobacco use.

## INTRODUCTION

Globally, tobacco causes approximately 6 million deaths each year, of which 80% occur in low- and middle-income countries^1,2^. Tobacco use is the most important modifiable risk factor for cancer and is among the leading preventable causes of death^1^. Thus, smoking prevention and cessation have a significant impact on health outcomes and are cost-effective interventions among a wide range of recommended evidence-based preventive health services^3^. As specified in the World Health Organization (WHO) Framework Convention on Tobacco Control (FCTC), a national comprehensive tobacco control program should include a systematic surveillance of tobacco use^4^. The Global Adult Tobacco Survey (GATS), using a standard protocol, is intended to support a country’s capacity to systematically monitor tobacco use in adults aged ≥15 years^5^.

Lao People’s Democratic Republic (Lao PDR) has made significant progress toward tobacco control in the past decade. In September 2006, Lao PDR became a full party to the WHO FCTC^6,7^. The National Assembly approved the National Tobacco Control Law in 2009, and the National Committee for Tobacco Control was established in 2012. The Lao Women’s Union and Ministry of Education and Sports have 100% smoke-free premises. Other ministries, including the Ministry of Health and Ministry of Public Works and Transport, are enforcing a smoke-free environment in their public facilities. Lao PDR is known for having instituted measures to prevent interference by the tobacco industry^8^. Nevertheless, areas for improvement in tobacco control remain. Tobacco tax (and price) in Lao PDR is still lower than that in other Southeast Asian countries^6,9,10^. In the most recent report available from WHO in 2017, no tobacco treatment program such as a toll-free quitline or nicotine replacement therapy was available or widely accessible in Lao PDR^10^.

In the 2012 Lao National Adult Tobacco Survey (NATS), 43.6% of men and 15.5% of women aged ≥15 years reported some type of tobacco use^7^. Data from other surveys suggested that tobacco use prevalence increased from 13.2% in 2007 to 18.7% in 2011 among male adolescents aged 13–15 years, and from 4.9% in 2007 to 6.0% in 2011 among female adolescents of the same age group^7^. It was estimated that tobacco-related diseases killed approximately 4800 Lao people in 2013, or approximately 13 people per day. The total national cost of inpatient health care for the three smoking-related diseases (stroke, lung cancer, and chronic obstructive pulmonary disease) was approximately $3.341 million in 2009, representing 0.8% of the gross domestic product and 22% of total health expenditure^7^. In summary, tobacco use is a burden in Lao PDR, and tobacco use surveillance is needed to inform appropriate policies and prevention strategies. This paper aims to report prevalence of various tobacco uses by selected sociodemographic characteristics, using data from the Lao NATS 2015.

## METHODS

The NATS followed the GATS standard protocol^5^. The NATS included all people aged ≥15 years, who considered Lao PDR their primary residence, and who were not institutionalized at the time of data collection. The NATS used a stratified 2-stage cluster sampling approach. Strata were 18 census-derived survey domains that represented 18 provinces. In each stratum, villages or comparable urban administrative units served as primary sampling units (PSU). At the first stage, PSUs were selected by using the probability proportional to size method. In each selected PSU, number of enumeration areas (EA) and number of households were obtained based on the national 2010 census. A list of households served as a sampling frame for the second stage, in which 20 households were selected from each PSU through a circular systematic sampling method. All eligible people aged ≥15 years in selected households were invited to participate. The response rate was 99.8% for household level and 85.0% for individual level, resulting in a total sample size of 7562 participants.

The Lao NATS 2015 questionnaire was based on the core questions from the GATS^5^, with additional country-specific questions. The questionnaire consisted of two parts: a household questionnaire, and an individual questionnaire. The individual questionnaire covered a wide range of related topics, including demographic characteristics, tobacco use by types, smoking characteristics, and awareness of harms caused by smoking. The questionnaires were pretested to ensure appropriate wording, comprehensibility, correct skip patterns, and appropriate interview time. CommCare software was used to administer and deliver the questions on tablets. Three interview teams collected the data. Each team consisted of one field supervisor and three interviewers. All staff were thoroughly trained to ensure standardization. The study received ethical review and approval from the Lao National Ethics Committee for Health Research.

The main variable for the analyses was current tobacco use. The standard GAST question was asked: ‘Do you currently smoke tobacco on a daily basis, less than daily, or not at all?’. A similar question was asked about smokeless tobacco use. Those who responded ‘daily’ or ‘less than daily’ were considered current tobacco users. Among tobacco users, a subsequent question elicited specific types of tobacco that they smoked or used. We used Stata 14.2 (StataCorp, College Station, TX) to perform the statistical analyses. All analyses were weighted for the complex survey design. We first performed basic descriptive statistics (percentages, means, medians) to examine variables of interest. We reported national estimates of overall and type-specific tobacco use by available sociodemographic characteristics, including gender, age, residence, and educational level. Multivariable logistic regression models were used to evaluate unadjusted and adjusted associations between independent and dependent variables of interest. We also examined interactions between sex and other sociodemographic characteristics for cigarette smoking. However, we could not do so for other types of tobacco uses, such as tobacco chewing in men, due to small observed numbers of cases. All p-values were two-tailed and were considered statistically significant if p<0.05.

## RESULTS

The NATS results showed that 32.4% of Lao people aged ≥15 years were current tobacco users (men: 51.2%, women: 15.4%) (Table 1). The prevalence of smoking any tobacco product was 27.9% (95% CI: 26.6–28.6) overall, 50.8% (95% CI: 46.9–52.2) in men, and 7.1% (95% CI: 6.3–7.9) in women. Cigarette smoking prevalence was 5.3% in women and 48.9% in men (prevalence ratio, PR=9.24, 95% CI: 6.80–12.56). Cigarette smoking accounted for approximately 95% of all tobacco use in men. Tobacco chewing, including betel quid, was common in women (9.1% among the general population, 60% among all female tobacco users, with PR=16.3, 95% CI: 9.4–28.2 when compared with men). Prevalence of tobacco chewing substantially increased in older age women, from approximately 1% in women aged <35 years to 9.2% aged 45–54 years and 32.3% for those aged ≥55 years.

**Table 1:** Prevalence of current tobacco use, weighted % (95% CI), Lao People’s Democratic Republic, 2015

*Characteristics*	*n*	*Total*	*All tobacco use*			*Cigarette smoking*			*Tobacco chewing*		
		*Weighted % (95% CI)*	*Total (n=2429)*	*Men (n=1833)*	*Women (n=596)*	*Total (n=1941)*	*Men (n=1743)*	*Women (n=198)*	*Total (n=359)*	*Men (n=17)*	*Women (n=342)*
Overall			32.4 (28.3–36.7)	51.2 (46.2–56.2)	15.4 (12.0–19.6)	26.0 (23.1–29.2)	48.9 (44.2–53.7)	5.3 (3.7–7.6)	5.1 (3.9–6.6)	0.6 (0.3–1.0)	9.1 (7.0–11.9)
**Age group (years)**											
15–24	1399	18.7 (17.4–20.0)	11.8 (8.9–11.6)	23.7 (17.5–31.3)	2.5 (1.4–4.5)	10.9 (8.2–14.3)	23.5 (17.4–31.0)	1.1 (0.3–3.4)	0.4 (0.2–0.7)	0.1 (0.0–1.1)	0.6 (0.3–1.1)
25–34	1589	20.9 (19.5–22.3)	22.7 (19.7–26.1)	43.2 (37.6–49.1)	6.3 (4.5–8.7)	20.3 (17.8–23.2)	41.5 (36.2–47.0)	3.3 (2.1–5.1)	0.7 (0.5–1.2)	0.0	1.3 (0.8–2.2)
35–44	1646	21.6 (20.4–22.8)	33.6 (28.7–38.8)	56.4 (50.6–62.1)	11.7 (8.2–16.4)	29.2 (25.2–33.5)	54.2 (48.5–59.8)	5.2 (3.5–7.7)	3.0 (2.0–4.5)	0.2 (0–1.1)	5.6 (3.7–8.4)
45–54	1464	19.2 (18.2–20.3)	40.1 (35.4–45.0)	62.4 (57.9–66.7)	20.5 (14.9–27.5)	33.0 (29.7–36.6)	60.3 (55.8–64.7)	9.0 (6.1–13.3)	5.1 (3.6–7.2)	0.5 (0.2–1.6)	9.2 (6.7–12.4)
≥55	1464	19.6 (18.1–21.2)	53.4 (47.3–59.6)	64.7 (59.5–69.5)	40.6 (32.4–49.2)	35.9 (32.1–39.9)	60.0 (55.4–64.4)	8.5 (5.4–13.1)	16.4 (13.1–20.5)	1.9 (1.1–3.3)	32.3 (25.9–41.0)
**Residence**											
Urban	2370	31.1 (11.6–60.9)	22.8 18.2–28.3)	40.3 (34.2–46.8)	7.5 (3.7–14.5)	19.4 (16.0–23.3)	39.3 (33.3–45.6)	1.9 (0.6–5.6)	3.1 (1.5–6.0)	0.3 (0.1–1.5)	5.5 (2.8–10.5)
Rural	5192	68.9 (39.1–88.4)	36.7 (33.3–40.2)	56.0 (52.6–59.3)	19.1 (15.2–23.6)	28.9 (26.4–31.6)	53.1 (49.5–56.8)	6.9 (4.8–9.7)	6.0 (4.5–7.9)	0.7 (0.5–1.1)	10.8 (8.0–14.5)
**Ethnicity**											
Lao	4542	61.1 (48.9–72.1)	29.9 (24.6–35.8)	48.8 (42.1–55.5)	13.3 (9.1–19.1)	24.7 (20.8–29.1)	48.1 (41.4–54.9)	4.3 (2.7–6.9)	5.2 (3.6–7.7)	0.3 (0.1–0.9)	9.6 (6.4–14.0)
Others^a^	3020	38.9 (27.9–51.1)	36.4 (32.4–40.5)	54.8 (50.1–59.4)	18.8 (14.5–24.1)	27.9 (24.6–31.5)	50.0 (45.0–55.1)	6.9 (4.5–10.4)	4.8 (3.8–6.0)	1.0 (0.7–1.5)	8.4 (6.6–10.7)
**Religion**											
Total	7561		2429	1833	596	1941	1743	198	359	17	342
Buddhist	5602	74.9 (63.8–83.4)	30.2 (25.6–35.4)	49.2 (43.2–55.1)	13.6 (9.8–18.5)	24.7 (21.3–28.5)	48.3 (42.5–54.1)	4.1 (2.7–6.4)	5.5 (3.9–7.5)	0.4 (0.2–1.0)	9.9 (7.1–13.7)
Others^b^	1800	22.2 (14.4–32.6)	38.7 (33.5–44.2)	56.6 (49.3–63.6)	20.9 (15.3–27.9)	29.5 (24.7–34.7)	50.5 (42.5–58.4)	8.5 (5.6–12.8)	4.2 (3.3–5.3)	1.2 (0.9–1.7)	7.2 (5.5–9.4)
None	159	1.7 (1.0–2.9)	42.4 (31.4–54.2)	61.1 (48.7–72.2)	25.8 (12.8–45.1)	32.8 (24.1–42.8)	53.3 (43.3–63.0)	14.6 (6.0–31.7)	0.0	0.0	0.0
**Marital status**											
Never married	1216	16.2 (13.7–19.2)	13.6 (10.9–16.8)	22.9 (18.6–28.0)	2.8 (1.4–5.5)	12.3 (9.8–15.4)	22.6 (18.1–27.8)	0.5 (0.1–1.9)	0.7 (0.4–1.3)	0.1 (0.0–1.0)	1.4 (0.7–2.6)
Currently married	5862	77.2 (74.3–79.9)	35.4 (31.6–39.4)	57.0 (52.6–61.4)	15.2 (11.8–19.3)	29.3 (26.5–32.2)	54.5 (50.4–58.6)	5.8 (4.1–8.1)	4.5 (3.3–6.0)	0.6 (0.3–1.0)	8.1 (6.0–10.8)
Divorced/separated/widowed	481	6.6 (5.8–7.3)	43.9 (37.1–51.0)	69.6 (58.6–78.7)	36.3 (29.9–43.4)	21.0 (15.4–27.9)	62.2 (52.0–71.4)	8.8 (5.4–14.0)	23.2 (19.2–27.6)	2.9 (0.6–14.0)	29.1 (24.0–34.9)
**Education level^c^**											
Never attended school	1260	16.9 (13.2–21.3)	48.4 (43.4–53.3)	71.7 (64.0–78.3)	40.2 (35.2–45.3)	25.8 (21.8–30.3)	62.8 (56.1–69.1)	12.9 (9.2–17.7)	18.8 (16.0–21.9)	2.3 (1.1–4.5)	24.6 (20.8–28.8)
Primary school	3027	39.6 (34.2–45.3)	35.7 (32.5–39.1)	61.4 (58.1–64.6)	13.1 (10.3–16.6)	30.2 (27.5–33.0)	58.4 (55.3–61.5)	5.3 (3.5–7.8)	4.0 (2.8–5.6)	0.5 (0.2–1.1)	7.1 (4.9–10.1)
Secondary school	1781	23.7 (22.4–25.1)	27.1 (23.9–30.5)	49.6 (44.3–54.9)	2.5 (1.6–3.7)	25.8 (22.9–29.0)	48.9 (43.6–54.2)	0.5 (0.2–1.4)	1.0 (0.7–1.5)	0.3 (0.1–1.1)	1.7 (1.2–2.6)
High school or higher	1494	19.8 (12.3–30.3)	18.4 (16.2–20.9)	29.7 (27.1–32.5)	0.8 (0.4–1.7)	17.9 (15.6–20.5)	29.2 (26.6–32.0)	0.2 (0.1–0.6)	0.5 (0.3–0.7)	0.3 (0.2–0.8)	0.7 (0.3–1.3)
**Income per day (US$)**											
<1.9^d^	1702	57.3 (48.1–66.1)	52.4 (49.0–55.9)	62.6 (58.9–66.0)	30.7 (25.9–36.1)	43.8 (40.5–47.1)	59.2 (54.7–63.5)	10.7 (7.5–15.1)	7.0 (5.4–9.0)	0.9 (0.5–1.7)	19.9 (15.5–25.2)
≥1.9	1251	42.7 (33.9–51.9)	40.9 (34.9–47.3)	56.6 (52.2–60.9)	9.7 (5.6–16.5)	37.2 (31.9–42.3)	54.4 (50.5–58.3)	2.8 (1.0–7.3)	3.0 (1.8–4.7)	0.6 (0.3–1.2)	7.7 (4.6–12.6)
**Occupation^e^**											
Unemployed	1264	16.9 (14.0–20.3)	23.2 (17.8–29.5)	29.7 (23.9–36.3)	19.4 (13.9–26.5)	14.0 (10.4–18.5)	27.3 (22.0–33.4)	6.4 (3.3–11.8)	8.4 (6.2–11.1)	1.0 (0.4–2.9)	12.5 (9.3–16.6)
Government sector	556	7.3 (5.5–9.6)	25.3 (21.6–29.4)	35.3 (30.2–40.7)	2.3 (0.9–5.5)	24.2 (20.2–28.8)	34.7 (29.4–40.4)	0.0	0.7 (0.3–1.7)	0.0	2.3 (0.9–5.5)
Non-government company/organization	1020	13.7 (10.3–18.1)	31.5 (24.5–39.5)	51.2 (42.4–60.0)	9.6 (5.8–15.9)	27.2 (21.7–33.5)	49.0 (41.0–57.1)	3.0 (1.4–6.4)	1.7 (1.0–2.9)	0.2 (0.0–1.8)	3.3 (1.9–5.8)
Agriculture	3755	50.2 (39.5–60.9)	37.8 (34.8–40.9)	60.4 (56.7–64.1)	17.3 (14.2–21.0)	30.6 (28.1–33.3)	57.3 (53.4–61.2)	6.6 (4.9–8.7)	5.4 (4.3–6.9)	0.8 (0.5–1.4)	9.6 (7.4–12.3)
Non-farm self-employed	433	5.6 (4.2–7.5)	34.9 (30.5–39.7)	55.1 (49.2–60.9)	6.4 (3.8–10.8)	32.6 (28.2–37.3)	55.1 (49.2–60.9)	0.8 (0.2–3.5)	2.3 (1.4–3.9)	0.0	5.6 (3.3–9.5)
Others	477	6.3 (4.4–8.8)	25.1 (17.8–34.1)	49.5 (42.8–56.2)	15.0 (7.8–26.7)	16.8 (13.5–20.8)	48.3 (41.1–55.6)	3.8 (2.4–6.0)	9.0 (4.5–17.2)	0.0	12.7 (6.1–24.4)

a Including PhouThai, Khermou, Khamu, Khmu, Leu, Mong etc.

b Including Christian, Pee, Phi, Phy, Pi etc.

c For those aged ≥18 years.

d International poverty line (http://povertydata.worldbank.org/poverty/country/LAO).

Supplementary Table 1 displays additional information about tobacco use in Lao PDR. Approximately 0.2% of men and 0.9% of women were concurrent users of smoked and smokeless tobacco. The prevalence of former tobacco users was 4.3% (men: 8.1%, women: 0.9%). Prevalence of former tobacco smokers was higher in the older age groups of men, <2% for those aged <35 years and ≥6% aged ≥45 years, and >9% in men who ever got married compared to 1.8% in men who never got married.

Bivariate associations between current tobacco use and selected demographic characteristics can be found in Supplementary Table 2. In the adjusted analysis (Table 2), current tobacco use was strongly associated with the older ages and lower education levels (p<0.001). For cigarette smoking, there appeared to be interactions between sex and education level and income. Specifically, women were more likely to have a lower education level and lower income than men, and these women were more likely to smoke. Prevalence of current cigarette smoking did not differ by urban versus rural residential area (p≥0.117), religious beliefs (p≥0.279), or marital status (p≥0.372) in either sex. The adjusted analysis confirmed that prevalence of tobacco chewing in women was significantly higher among the elder women. Tobacco chewing in women was also associated with lower education level and lower income level. Compared to women who attended secondary school or higher, women who never attended school were five times more likely to chew tobacco (odds ratio, OR=5.7, 95% CI: 3.0–10.9).

**Table 2:** Adjusted associations, OR (95% CI), between current tobacco use (versus never/former used) and selected characteristics, Lao People’s Democratic Republic, 2015

*Characteristics*	*All tobacco use*		*Cigarette smoking*		p**	*Tobacco chewing in women*
	*Men*	*Women*	*Men*	*Women*		
	*Current=1833 *Never/Former=1752	*Current=596 Never/Former=3381	*Current=1743 Never/Former=1842	*Current=198 Never/Former=3779		*Current=342 Never/Former=3635
**Age group (years)**						
15–24	1	1	1	1		–^e^
25–34	0.98 (0.52–1.83)	1.23 (0.34–4.47)	0.86 (0.45–1.65)	0.88 (0.25–3.04)		1
35–44	1.32 (0.74–2.37)	1.97 (0.48–8.10)	1.14 (0.60–2.16)	1.15 (0.27–4.88)		4.09 (1.83–9.15)
45–54	1.70 (0.97–2.98)	3.40 (0.88–13.20)	1.49 (0.83–2.69)	1.84 (0.35–9.69)		5.05 (2.16–11.81)
≥55	1.74 (0.97–3.15)	7.66 (1.94–30.30)	1.38 (0.73–2.60)	1.49 (0.41–5.38)		17.95 (8.35–38.61)
p (p-trend)	<0.001 (<0.001)	<0.001 (<0.001)	<0.001 (<0.001)	0.552 (0.161)	0.985	<0.001 (<0.001)
**Residence**						
Urban	1	1	1	1		1
Rural	1.40 (1.01–1.94)	1.71 (0.75–3.88)	1.33 (0.93–1.91)	1.89 (0.58–6.13)		1.50 (0.60–3.78)
p	0.044	0.212	0.117	0.278	0.495	0.371
**Ethnicity**						
Lao	1	1	1	1		1
Others^a^	0.76 (0.55–1.04)	0.89 (0.43–1.85)	0.72 (0.53–0.97)	0.94 (0.47–1.86)		0.70 (0.27–1.80)
p	0.086	0.745	0.032	0.848	0.392	0.438
**Religion**						
Buddhist	1	1	1	1		1
Others^b^	1.40 (0.89–2.22)	1.70 (0.90–3.19)	1.13 (0.69–1.89)	2.28 (0.80–6.50)		0.57 (0.22–1.47)
None	1.67 (0.92–3.03)	1.51 (0.12–18.24)	1.33 (0.82–2.14)	3.28 (0.39–27.75)		–
p	0.190	0.251	0.495	0.279	0.423	0.232
**Marital status**						
Never married	1.48 (0.82–2.69)	1.84 (0.50–6.78)	1.49 (0.84–2.63)	0.78 (0.08–7.83)		0.93 (0.15–5.72)
Currently married	1	1	1	1		1
Divorced/ separated/ widowed	1.15 (0.78–2.69)	1.34 (0.81–2.21)	1.00 (0.68–1.48)	0.97 (0.57–1.63)		1.54 (0.91–2.61)
p	0.396	0.326	0.372	0.967	0.821	0.274
**Education level^c^**						
Never attended school	2.58 (1.67–3.98)	9.10 (4.44–18.65)	2.02 (1.43–2.85)	24.70 (3.03–201.22)		5.69 (2.97–10.89)
Primary school	1.86 (1.29–2.68)	2.64 (1.07–6.50)	1.82 (1.30–2.53)	15.37 (1.51–155.92)		1.60 (0.58–4.42)
Secondary school	1.51 (1.07–2.15)	1	1.57 (1.12–2.20)	1		1
High school or higher	1	–^f^	1	–^f^		–^f^
p (p-trend)	<0.001 (<0.001)	<0.001 (<0.001)	<0.001 (<0.001)	<0.001 (<0.001)	0.055	<0.001
**Income per day (US$)**						
<1.9^d^	1.06 (0.91–1.24)	2.51 (1.44–4.38)	1.11 (0.94–1.31)	2.21 (1.12–4.37)		2.06 (1.28–3.30)
≥1.9	1	1	1	1		1
p	0.449	0.002	0.223	0.029	0.048	0.004

a Including PhouThai, Khermou, Khamu, Khmu, Leu, Mong etc.

b Including Christian, Pee, Phi, Phy, Pi etc.

c For those aged ≥18 years.

d International poverty line (http://povertydata.worldbank.org/poverty/country/LAO).

e This age group was merged with the 25–34 years age group due to very low or zero frequencies.

f This education level was merged with secondary school for analyses in women due to very low or zero frequencies.

** p-value for interaction by sex.

* Unweighted counts.

Among current cigarette smokers (Table 3), 92.3% smoked daily. Of these, the mean age at which respondents began the daily smoking habit was 17.4 years. About one-third (32%) of current smokers used hand-rolled cigarettes, alone or in combination with manufactured cigarettes. People living in rural areas were approximately five times more likely to use hand-rolled cigarettes than were people living in urban areas (OR=5.7, 95% CI: 3.7–9.0 for men, and OR=4.2, 95% CI: 1.8–9.9 for women).

**Table 3:** Smoking characteristics, weighted % (95% CI), among current cigarette smokers, Lao People’s Democratic Republic, 2015

*Characteristics*	*Total (n=1941)*	*Men*			*Women*		
		*Total (n=1743)*	*Urban (n=437)*	*Rural (n=1306)*	*Total (n=198)*	*Urban (n=29)*	*Rural (n=169)*
**Smoking frequency**							
Daily	92.3 (91.0–93.4)	92.4 (90.9–93.6)	89.8 (86.1–92.6)	93.2 (91.8–94.4)	91.6 (87.7–94.3)	85.7 (70.4–93.9)	92.3 (87.3–95.5)
Occasionally	7.7 (6.6–9.2)	7.6 (6.4–9.1)	10.2 (7.4–13.9)	6.8 (5.6–8.2)	8.4 (5.7–12.3)	14.3 (6.2–29.6)	7.7 (4.5–12.7)
**Types of cigarette product**							
Manufactured only	68.1 (62.7–73.1)	73.2 (68.4–77.5)	88.9 (84.1–92.4)	68.1 (63.2–72.6)	25.1 (17.0–35.5)	57.2 (39.6–73.1)	20.9 (12.3–33.4)
Hand-rolled only	23.6 (19.3–28.4)	18.5 (14.9–22.8)	5.0 (3.0–8.3)	22.9 (28.9–27.5)	66.5 (56.6–75.1)	33.9 (16.8–56.6)	70.8 (57.7–81.1)
Both manufactured and hand-rolled	8.3 (6.5–10.5)	8.3 (6.4–10.6)	6.1 (3.7–9.9)	9.0 (6.7–11.9)	8.4 (6.5–10.7)	8.9 (3.3–21.8)	8.3 (6.2–11.1)
**Cigarettes per day**							
Median (Q1–Q3)	10 (6–20)	10 (7–20)	10 (10–20)	10 (7–20)	7 (4–10)	8 (3–10)	7 (4–10)
**Spending on cigarettes in past week^a^ (US$)**							
Median (Q1–Q3)	1.2 (0.0–2.4)	1.4 (0.4–2.5)	1.7 (0.5–3.4)	1.2 (0.2–2.4)	0.2 (0.0–1.4)	2.4 (1.2–4.2)	0.0 (0.0–1.2)
**Per cent of income on cigarettes in past week**							
Mean (SD)	11.7 (1.0)	11.9 (1.0)	11.9 (1.5)	11.8 (0.6)	8.0 (2.3)	6.7 (2.3)	8.2 (1.1)
**Minutes to first cigarette after waking^b^**							
<5	15.7 (13.0–18.9)	15.8 (12.9–19.2)	18.9 (10.4–32.0)	14.8 (12.1–18.0)	15.4 (9.7–23.4)	14.8 (5.4–34.7)	15.4 (9.2–24.9)
5–30	24.7 (21.6–28.0)	25.3 (22.1–28.8)	29.5 (24.3–35.2)	24.0 (20.0–28.6)	19.3 (13.6–26.7)	11.1 (4.7–24.1)	20.3 (14.3–27.9)
31–60	13.7 (11.8–15.8)	13.6 (11.6–15.8)	18.2 (13.2–24.6)	12.1 (9.5–15.2)	14.4 (11.9–17.4)	21.8 (11.8–36.8)	13.6 (11.2–16.4)
>60	45.9 (41.6–50.4)	45.3 (40.8–50.0)	33.4 (24.0–44.4)	49.1 (45.2–53.1)	50.9 (44.2–57.5)	52.3 (34.3–69.7)	50.7 (43.9–57.5)
**Ever been advised to quit by a healthcare provider^c^**							
No	80.5 (75.6–84.7)	82.1 (76.2–86.8)	79.1 (69.1–86.4)	83.1 (77.6–87.4)	70.8 (59.5–80.0)	62.5 (27.1–88.1)	72.0 (58.4–82.5)
Yes	19.5 (15.3–24.4)	17.9 (13.2–23.8)	20.9 (13.6–30.9)	16.9 (12.6–22.4)	29.2 (20.0–40.5)	37.5 (11.9–72.9)	28.0 (17.6–41.6)
**Interested in quitting smoking**							
Plan to quit within next month	3.9 (2.7–5.8)	4.0 (2.7–5.8)	5.2 (3.0–8.8)	3.6 (2.1–6.1)	3.7 (1.7–7.8)	3.9 (1.1–13.1)	3.7 (1.6–8.2)
Think about quitting within next 12 months	4.4 (3.2–5.8)	4.2 (3.2–5.6)	5.5 (3.2–9.3)	3.9 (2.7–5.4)	4.9 (2.5–9.5)	5.3 (0.6–33.8)	4.9 (2.2–10.4)
Will quit someday, but not in the next 12 months	19.1 (15.4–23.5)	19.9 (15.8–24.8)	29.5 (18.8–43.1)	16.9 (14.4–19.9)	12.3 (6.2–23.0)	11.3 (2.3–40.9)	12.4 (6.5–22.5)
Not interested in quitting	72.6 (67.3–77.4)	71.9 (66.1–77.0)	59.8 (44.3–73.6)	75.6 (73.1–78.0)	79.1 (67.5–87.3)	79.5 (55.4–92.4)	79.0 (67.2–87.4)

Q: quartile; SD: standard deviation; n: unweighted counts.

a Among manufactured and hand-rolled cigarette (local business-made or home-made) users.

b Among daily cigarette smokers. Cigarettes include manufactured and hand-rolled types.

c Among those who visited healthcare provider.

The top five manufactured cigarette brands purchased in the last month included Adeng (white soft package, 44%), Adeng Full Flavor (hard package, 22%), Dokmaideng (15%), Adeng Menthol (6%), and Jonnee Green (6%). Most manufactured cigarettes were purchased at grocery/convenience stores (77%) and at traditional street markets (18%). The median numbers of cigarettes smoked per day were 10 for all male smokers, 7 for all female smokers, 14 for daily male smokers, and 8 for daily female smokers. Proportions of income spent on cigarettes in the past week were 12% for men and 8% for women. About 40% of smokers smoked the first cigarette in ≤30 minutes after waking up daily.

Most smokers (80%) had never received advice to quit smoking from a healthcare provider. The majority of current smokers (88.4%) also believed that smoking causes bronchitis, lung cancer, or heart diseases. This rate was not statistically different from that in non-smokers (Supplementary Table 3). Nevertheless, only 3.9% of current smokers planned to quit in the next month, while 4.4% thought about quitting in the next 12 months.

## DISCUSSION

This NATS report in Lao PDR provides national adjusted estimates for different types of tobacco uses by selected sociodemographic characteristics. Policy-makers can use this information to strengthen national tobacco control strategies. The prevalence of tobacco smoking in Lao PDR is comparable to that in Myanmar (26.1%) but is higher than the smoking prevalence in other neighboring countries, such as 19.1% in Thailand, 16.9% in Cambodia, and 22.5% in Vietnam^9^. Of these countries, Thailand has been the most successful in implementing WHO MPOWER measures for tobacco control, including monitoring, smoke-free policies, cessation programs, health warnings, mass media, advertising bans, and taxation^11^. Lao PDR has performed as well as Thailand and equally or better than Myanmar with regard to enforcing smoke-free policies, requiring health warnings on cigarette packaging, and banning tobacco advertisements^10,12^. However, tobacco smoking prevalence in Lao PDR is still high, perhaps due to the low rate of cigarette tax. Compared with the same NATS data in Lao PDR in 2012, the prevalence of all-tobacco use slightly increased in men (51.2% in 2015 vs 43.6% in 2012) and remained similar in women (15.4% in 2015 vs 15.5% in 2012)^7^. As of the end of 2017, no treatment program for tobacco dependence was available in Lao^10,13^. Providing tobacco cessation treatment is necessary not only to reduce the national tobacco use rate but also to reduce smoking-related morbidity and mortality rates. Moreover, although cigarettes were the most common form of tobacco use in Lao PDR, electronic nicotine delivery systems (i.e. e-cigarettes) have been increasingly promoted and sold in the region^14^. Therefore, future NATS or other tobacco surveillances should monitor e-cigarette use to inform national tobacco control strategies.

The 2015 Lao NATS data revealed variations in types and prevalence of tobacco use across sociodemographic subpopulations. Cigarette smoking was the main form of tobacco use in men, while tobacco chewing was much more common in women. This difference by sex was similar to observations in several south and southeast Asian countries^9,15,16^. To reduce tobacco use prevalence in Lao PDR, tobacco treatment programs must take into account the diversity of tobacco usage, and must be gender-specific.

Of note is the vicious interaction between tobacco dependence and poverty. The NATS results showed that tobacco use prevalence was higher in populations with lower income and lower education level, which presumably led to an even lower income. Spending on cigarettes in the past week accounted for approximately 12% of smokers’ family income. In a large national sample of Cambodian adults, tobacco use was highest in those earning less than US$2 per day^17^. Conditions of extreme poverty did not appear to influence tobacco users to decrease their use of smoked or smokeless tobacco^18^. Tobacco spending detracts from total household income, reducing the amount that is available to spend on essential household needs. Furthermore, approximately 88% of hospitalization costs due to tobacco-related diseases in Lao PDR were mainly paid by smokers or their families^19^. Tobacco treatment programs may primarily target low socioeconomic status populations to alleviate the affliction of tobacco dependence in the poor.

Tobacco use generally increased in older age groups. This finding is similar to a general pattern observed in other Asian countries^16,17^. When Laotians reach working age, they may be influenced by their co-workers to smoke, given the culture that offering cigarettes is an acceptable social norm to show one’s respect or friendliness, and to develop and maintain business relationships^20-22^. Additionally, older people may have more financial independence or more autonomy to purchase tobacco. Although cigarette smoking is less prevalent among the youth, e-cigarette marketing to young people has grown extensively and e-cigarette use among adolescents has increased at phenomenal rates globally^14,23^. Therefore, the government should be prepared for this next wave of nicotine dependence control.

Most smokers in this NATS had never received advice to quit smoking from a healthcare provider. Another survey indicated that most medical doctors lacked experience in providing counselling about the adverse health effects caused by tobacco and how to quit smoking^24^. A hospital visit or admission provides a great opportunity for smoking cessation treatment, especially when the illness that brings smokers to the healthcare facilities is caused by or related to tobacco use^25^. Although offering smoking cessation treatment is now standard care in several hospitals in developed countries, there is no such care available in hospitals in Lao PDR. Most tobacco smokers were aware that smoking causes diseases such as bronchitis, lung cancer, or heart diseases; yet, they still smoked. This finding suggests that simply raising awareness, either through brief advice from healthcare providers or through health warnings on cigarette packages, is not sufficient to help smokers quit. Comprehensive tobacco treatments that include a pharmacological component such as nicotine replacement therapy and behavioral interventions may be necessary to advance smoking cessation rates.

### Limitations

Although the NATS comprised a nationally representative sample, it had limitations. Tobacco use was assessed via self-reports without biochemical confirmation; thus, actual use might have been under-reported. Some specific institutionalized populations were not included. Due to the cross-sectional nature of the data, temporal relationships cannot be inferred. Some specific types of tobacco or nicotine uses were not mentioned or were separately assessed. In addition, some subgroups had low frequencies, resulting in wide confidence intervals in analyses.

## CONCLUSIONS

Findings from the 2015 NATS showed that tobacco use in Lao PDR was prevalent and was among the highest in the region. Most tobacco use in Lao PDR was cigarette smoking, but tobacco chewing was also common in women. The government should continue current national tobacco control efforts and implement additional proven strategies to reduce tobacco use. Different evidence-based cessation interventions tailored to different subgroups are needed to reduce tobacco use and tobacco-related morbidity rates. These tobacco treatment programs must take into account the diversity of tobacco usage and must be gender-specific. If resources for tobacco treatment are limited, treatment programs should primarily target low socioeconomic status populations to alleviate the afflictions of tobacco dependence.
