# Supplementary material for: Tobacco use in Lao People’s Democratic Republic: Results from the 2015 National Adult Tobacco Survey
Source: Tob Prev Cessat. 2019 Sep 30;5:31. doi: 10.18332/tpc/112248 (PMC7205112; doi:10.18332/tpc/112248)
Supplement: Supplementary file 1 [file TPC-5-31-s1.pdf]

Table S1: Prevalence of other tobacco uses in Lao People's Democratic Republic, 2015, weighted prevalence (95% CI)

| Characteristics | Other smoked tobacco current users[a] | | | Current users of both smoked and smokeless tobacco | | | Former tobacco smokers | | |
|---|---|---|---|---|---|---|---|---|---|
| | Total (*n*=206) | Men (*n*=115) | Women (*n*=91) | Total (*n*=36) | Men (*n*=6) | Women (*n*=30) | Total (*n*=319) | Men (*n*=283) | Women (*n*=36) |
| Overall, % (95% CI) | 2.4 (1.6-3.7) | 2.9 (1.8-4.5) | 2.0 (1.1-3.7) | 0.6 (0.3-1.1) | 0.2 (0.1-0.5) | 0.9 (0.5-1.8) | 4.3 (3.2-5.7) | 8.1 (5.8-11.2) | 0.9 (0.6-1.2) |
| Age group (years) | | | | | | | | | |
| 15-24 | 0.7 (0.3-1.5) | 0.3 (0.1-1.6) | 0.9 (0.4-2.1) | 0.1 (0.0-0.5) | 0.1 (0.0-1.1) | 0.0 | 0.9 (0.7-1.2) | 1.9 (1.5-2.5) | 0.1 (0.0-0.6) |
| 25-34 | 2.2 (1.3-3.7) | 2.9 (1.7-5.0) | 1.6 (0.8-3.4) | 0.0 | 0.0 | 0.0 | 1.9 (1.0-3.6) | 3.9 (1.9-7.9) | 0.3 (0.1-1.0) |
| 35-44 | 2.9 (1.7-4.7) | 3.4 (1.9-6.0) | 2.4 (1.3-4.3) | 0.7 (0.3-1.5) | 0.1 (0.0-1.1) | 1.2 (0.5-2.8) | 4.1 (3.0-5.5) | 7.4 (5.3-10.1) | 1.0 (0.5-1.7) |
| 45-54 | 3.1 (1.8-5.1) | 3.0 (1.6-5.5) | 3.1 (1.5-6.3) | 0.5 (0.2-1.7) | 0.3 (0.1-1.4) | 0.7 (0.3-2.0) | 6.0 (4.7-7.7) | 11.3 (8.3-15.2) | 1.4 (0.8-2.4) |
| ≥55 | 3.2 (2.3-4.7) | 4.3 (2.8-6.6) | 2.1 (1.0-4.2) | 1.6 (0.9-2.8) | 0.4 (0.1-0.9) | 3.0 (1.6-5.5) | 8.5 (6.0-11.9) | 14.6 (9.9-21.0) | 1.7 (1.2-2.4) |
| Residence | | | | | | | | | |
| Urban | 0.7 (0.3-1.5) | 1.1 (0.5-2.5) | 0.3 (0.1-1.2) | 0.1 (0.0-0.6) | 0.0 | 0.2 (0.1-1.2) | 5.5 (3.3-8.8) | 11.1 (6.5-18.2) | 0.5 (0.2-1.2) |
| Rural | 3.2 (2.0-5.1) | 3.7 (2.3-6.0) | 2.8 (1.4-5.4) | 0.8 (0.4-1.5) | 0.3 (0.1-0.6) | 1.2 (0.6-2.5) | 3.8 (3.0-4.7) | 6.8 (5.3-8.6) | 1.0 (0.7-1.5) |
| Ethnicity | | | | | | | | | |
| Lao | 0.9 (0.6-1.4) | 1.3 (0.7-2.4) | 0.6 (0.4-0.8) | 0.6 (0.3-1.5) | 0.2 (0.0-0.6) | 1.0 (0.4-2.5) | 4.6 (3.2-6.5) | 8.9 (5.9-13.3) | 0.8 (0.4-1.5) |
| Others[b] | 4.8 (3.0-7.6) | 5.3 (3.0-9.0) | 4.4 (2.3-8.3) | 0.5 (0.3-0.8) | 0.3 (0.1-0.5) | 0.7 (0.4-1.2) | 3.8 (2.9-5.1) | 6.8 (4.9-9.4) | 1.0 (0.6-1.8) |
| Religion | | | | | | | | | |
| Buddhist | 0.9 (0.6-1.3) | 1.3 (0.8-2.3) | 0.5 (0.4-0.7) | 0.6 (0.2-1.4) | 0.1 (0.0-0.5) | 0.9 (0.4-2.3) | 4.8 (3.6-6.4) | 9.6 (6.9-13.1) | 0.7 (0.4-1.3) |
| Others | 6.3 (4.2-9.5) | 6.6 (3.6-11.6) | 6.1 (3.1-11.6) | 0.7 (0.5-1.0) | 0.4 (0.2-0.9) | 1.0 (0.6-1.4) | 2.4 (1.7-3.3) | 3.8 (2.6-5.4) | 1.0 (0.5-2.2) |
| None | 15.9 (6.2-34.9) | 16.7 (6.9-35.1) | 15.2 (5.1-37.2) | 0.0 | 0.0 | 0.0 | 5.8 (3.1-10.9) | 6.2 (2.1-17.0) | 5.5 (3.6-8.4) |
| Marital status | | | | | | | | | |
| Never married | 0.8 (0.3-2.1) | 0.7 (0.2-1.8) | 1.1 (0.3-4.2) | 0.1 (0.0-0.6) | 0.1 (0.0-1.0) | 0.0 | 0.9 (0.3-2.6) | 1.8 (0.6-4.8) | 0.0 |
| Currently married | 2.8 (1.9-4.1) | 3.3 (2.2-5.1) | 2.3 (1.3-3.9) | 0.5 (0.2-1.1) | 0.2 (0.1-0.6) | 0.8 (0.4-1.8) | 5.0 (3.8-6.7) | 9.5 (6.8-13.1) | 0.9 (0.6-1.4) |
| Divorced/ Separated/ Widowed | 2.4 (1.2-4.5) | 5.1 (2.0-12.5) | 1.6 (0.7-3.5) | 2.4 (1.5-4.0) | 0.0 | 3.1 (1.8-5.3) | 3.9 (2.5-5.9) | 10.1 (5.7-17.3) | 2.0 (1.2-3.4) |
| Education level[c] | | | | | | | | | |
| Never attended school | 6.6 (4.1-10.5) | 9.6 (6.0-15.0) | 5.6 (2.8-10.7) | 2.0 (1.1-3.5) | 0.5 (0.3-0.8) | 2.5 (1.4-4.6) | 2.8 (2.0-3.7) | 5.9 (4.2-8.2) | 1.7 (1.1-2.4) |
| Primary school | 2.7 (1.7-4.3) | 4.0 (2.2-6.9) | 1.6 (0.9-2.8) | 0.5 (0.2-1.3) | 0.2 (0.1-0.7) | 0.8 (0.3-1.9) | 4.3 (3.3-5.8) | 8.2 (5.9-11.2) | 0.9 (0.6-1.5) |
| Secondary school | 0.7 (0.4-1.3) | 1.2 (0.7-2.2) | 0.2 (0.1-0.4) | 0.1 (0.0-0.6) | 0.1 (0.0-1.1) | 0.0 | 4.5 (3.1-6.5) | 8.4 (5.7-12.1) | 0.4 (0.1-1.6) |
| High school or higher | 0.4 (0.2-0.8) | 0.6 (0.3-1.3) | 0.0 | 0.1 (0.0-0.5) | 0.1 (0.0-0.8) | 0.0 | 5.2 (3.6-7.4) | 8.4 (5.3-13.2) | 0.2 (0.0-1.4) |

| | | | | | | | | | |
|---|---|---|---|---|---|---|---|---|---|
| Income per day in US dollars | | | | | | | | | |
| <1.9[d] | 3.7 (2.1-6.2) | 4.1 (2.3-7.4) | 2.7 (1.1-6.5) | 1.2 (0.7-2.0) | 0.5 (0.2-1.3) | 2.5 (1.4-4.7) | 6.5 (5.1-8.3) | 8.7 (6.6-11.4) | 1.8 (1.1-2.8) |
| ≥1.9 | 1.5 (0.9-2.5) | 2.0 (1.3-3.3) | 0.3 (0.0-2.4) | 0.4 (0.1-1.2) | 0.0 | 1.1 (0.3-3.9) | 8.6 (6.1-22.0) | 12.7 (8.6-18.3) | 0.5 (0.1-2.2) |

*Note:* n: unweighted counts

[a] Including cigars, tobacco pipe, and water-pipes

[b] including PhouThai, Khermou, Khamu, Khmu, Leu, Mong, etc.

[c] for those aged ≥18

[d] International poverty line (http://povertydata.worldbank.org/poverty/country/LAO)

Table S2: Bivariate associations between current tobacco use (versus never/former used) and selected characteristics, Lao People's Democratic Republic, 2015, OR (95% CI)

| Characteristics | All tobacco use | | Cigarette smoking | | Tobacco chewing in women |
|---|---|---|---|---|---|
| | Men | Women | Men | Women | |
| Unweighted counts | Current=1833, Never/Former=1752 | Current=596, Never/Former=3381 | Current=1743 Never/Former=1842 | Current=198 Never/Former=3779 | Current=342 Never/Former=3635 |
| Age group (years) | | | | | |
| 15-24 | 1 | 1 | 1 | 1 | – [e] |
| 25-34 | 2.45 (1.83-3.27) | 2.61 (1.74-3.90) | 2.31 (1.73-3.09) | 3.19 (1.33-7.66) | 1 |
| 35-44 | 4.17 (3.21-5.41) | 5.17 (3.10-8.64) | 3.85 (2.92-5.08) | 5.13 (1.75-15.03) | 6.12 (3.96-9.48) |
| 45-54 | 5.34 (3.95-7.20) | 10.05 (6.51-15.53) | 4.95 (3.68-6.66) | 9.25 (3.58-23.89) | 10.48 (6.41-17.15) |
| ≥55 | 5.88 (4.34-7.97) | 26.61 (14.73-48.06) | 4.88 (3.53-6.75) | 8.69 (3.12-24.19) | 53.08 (35.55-79.26) |
| *P* trend | <.001 | <.001 | <.001 | <.001 | <.001 |
| Residence | | | | | |
| Urban | 1 | 1 | 1 | 1 | 1 |
| Rural | 1.88 (1.35-2.63) | 2.92 (1.18-7.24) | 1.75 (1.25-2.46) | 3.80 (1.04-13.86) | 2.08 (0.80-5.37) |
| Ethnicity | | | | | |
| Lao | 1 | 1 | 1 | 1 | 1 |
| Others[a] | 1.27 (0.94-1.72) | 1.51 (0.88-2.58) | 1.08 (0.78-1.50) | 1.64 (0.90-3.00) | 0.87 (0.53-1.44) |
| Religion | | | | | |
| Buddhist | 1 | 1 | 1 | 1 | 1 |
| Others[b] | 1.35 (0.93-1.96) | 1.68 (0.99-2.86) | 1.09 (0.73-1.63) | 2.16 (1.18-3.97) | 0.65 (0.40-1.05) |
| None | 1.63 (0.96-2.77) | 2.21 (0.86-5.70) | 1.22 (0.80-1.88) | 3.98 (1.46-10.86) | – |
| Marital status | | | | | |
| Never married | 0.22 (0.18-0.28) | 0.26 (0.09-0.29) | 0.24 (0.19-0.31) | 0.08 (0.02-0.30) | 0.16 (0.09-0.26) |
| Currently married | 1 | 1 | 1 | 1 | 1 |
| Divorced/ Separated/ Widowed | 1.72 (1.15-2.57) | 3.19 (2.59-3.93) | 1.37 (0.97-1.93) | 1.57 (1.11-2.23) | 4.67 (3.60-6.07) |
| Education level[c] | | | | | |
| Never attended school | 6.00 (4.16-8.65) | 36.86 (26.67-50.96) | 4.10 (3.08-5.44) | 38.10 (19.81-73.27) | 25.47 (18.04-35.96) |
| Primary school | 3.76 (3.12-4.53) | 8.28 (6.01-11.40) | 3.40 (2.88-4.02) | 14.30 (5.74-35.60) | 5.81 (3.82-8.85) |
| Secondary school | 2.33 (1.88-2.89) | 1 | 2.32 (1.86-2.88) | 1 | 1 |
| High school or higher | 1 | – [f] | 1 | – [f] | – [f] |
| *P* trend | <.001 | <.001 | <.001 | <.001 | <.001 |
| Income per day in US dollars | | | | | |
| <1.9[d] | 1.28 (1.04-1.57) | 4.12 (2.43-6.97) | 1.21 (0.97-1.51) | 4.12 (1.72-9.89) | 2.99 (1.89-4.73) |

| | | | | | |
|---|---|---|---|---|---|
| ≥1.9 | 1 | 1 | 1 | 1 | 1 |
| Occupation | | | | | |
| Unemployed | 1 | 1 | 1 | 1 | 1 |
| Government sector | 1.29 (0.99-1.68) | 0.10 (0.03-0.32) | 1.41 (1.08-1.85) | - | 0.16 (0.05-0.49) |
| Non-government company/organization | 2.48 (1.88-3.29) | 0.44 (0.33-0.59) | 2.56 (1.96-3.33) | 0.45 (0.34-0.61) | 0.24 (0.17-0.34) |
| Agriculture | 3.61 (2.82-4.63) | 0.87 (0.59-1.28) | 3.57 (2.77-4.61) | 1.03 (0.56-1.92) | 0.74 (0.53-1.03) |
| Non-farm self-employed | 2.91 (1.98-4.26) | 0.28 (0.16-0.51) | 3.27 (2.28-4.67) | 0.12 (0.02-0.59) | 0.42 (0.22-0.78) |
| Others | 2.32 (1.72-3.13) | 0.73 (0.48-1.11) | 2.49 (1.86-3.32) | 0.59 (0.34-1.00) | 1.01 (0.59-1.74) |

[a] including PhouThai, Khermou, Khamu, Khmu, Leu, Mong, etc.

[b] including Christian, Pee, Phi, Phy, Pi, etc.

[c] for those aged ≥18

[d] International poverty line (http://povertydata.worldbank.org/poverty/country/LAO)

[e] This age group was merged with the 25–34 year age group due to very low or zero frequencies.

[f] This education level was merged with secondary school for analyses in women due to very low or zero frequencies.

Table S3: Percentage of adults aged ≥15 years who believed that smoking causes bronchitis, lung cancer, or heart diseases, by smoking status and selected demographic characteristics, Lao People's Democratic Republic, 2015, weighted % (95% CI)

| Characteristics | Overall | Current tobacco smokers | Never tobacco smokers | *P* value[d] |
|---|---|---|---|---|
| Overall | **90.4 (87.8-92.4)** | **88.4 (85.6-90.7)** | **90.8 (87.8-93.0)** | .083 |
| Sex | | | | |
| Male | 92.6 (90.5-94.3) | 90.8 (88.5-92.6) | 94.0 (91.2-95.9) | .007 |
| Female | 88.3 (85.1-91.0) | 73.1 (66.3-79.0) | 89.5 (86.2-92.0) | <.001 |
| Age group (years) | | | | |
| 15-24 | 92.9 (91.2-94.3) | 90.8 (87.5-93.3) | 93.2 (91.3-94.8) | .145 |
| 25-34 | 89.9 (86.6-92.5) | 87.4 (83.6-90.5) | 90.5 (86.6-93.4) | .102 |
| 35-44 | 90.6 (87.7-92.9) | 89.1 (85.2-92.1) | 91.0 (87.4-93.7) | .303 |
| 45-54 | 90.7 (87.8-93.0) | 89.9 (87.0-92.2) | 90.5 (86.6-93.3) | .733 |
| ≥55 | 87.8 (83.8-90.9) | 86.3 (81.9-89.8) | 87.1 (81.5-91.1) | .772 |
| Residence | | | | |
| Urban | 95.0 (91.3-97.2) | 93.5 (90.6-95.5) | 95.4 (90.8-97.7) | .280 |
| Rural | 88.2 (85.1-90.8) | 86.9 (82.8-90.2) | 88.3 (85.2-90.9) | .287 |
| Ethnicity | | | | |
| Lao | 94.7 (93.0-96.0) | 93.6 (92.0-94.8) | 94.9 (92.7-96.5) | .210 |
| Others[a] | 83.4 (80.9-85.7) | 81.9 (77.9-85.2) | 83.5 (80.7-85.9) | .369 |
| Religion | | | | |
| Buddhist | 93.9 (92.1-95.3) | 93.1 (91.4-94.4) | 93.9 (91.6-95.7) | .424 |
| Others[b] | 78.5 (75.0-81.7) | 76.9 (72.0-81.2) | 78.8 (74.4-82.7) | .469 |
| None | 95.8 (92.3-97.7) | 94.7 (82.1-98.6) | 96.1 (92.0-98.2) | .498 |
| Education level[c] | | | | |
| Never attended school | 72.0 (67.6-76.0) | 69.4 (62.2-75.8) | 72.4 (67.9-76.5) | .369 |
| Primary school | 91.5 (89.0-93.4) | 90.9 (88.6-92.8) | 91.4 (88.4-93.6) | .711 |
| Secondary school | 95.2 (94.0-96.2) | 95.2 (93.4-96.6) | 95.0 (93.3-96.2) | .790 |
| High school or higher | 97.8 (97.0-98.4) | 95.6 (92.1-97.5) | 98.3 (97.7-98.8) | .008 |

[a] including PhouThai, Khermou, Khamu, Khmu, Leu, Mong, etc.
[b] including Christian, Pee, Phi, Phy, Pi, etc.
[c] for those aged ≥18 years
[d] For the difference in prevalence in current tobacco smokers versus in never tobacco smokers, using Pearson Chi-square test.

© 2019 Xangsayarath P.
